# Cushing’s Syndrome and Hypothalamic–Pituitary–Adrenal Axis Hyperactivity in Chronic Central Serous Chorioretinopathy

**DOI:** 10.3389/fendo.2018.00039

**Published:** 2018-02-20

**Authors:** Femke M. van Haalen, Elon H. C. van Dijk, Olaf M. Dekkers, Maurice B. Bizino, Greet Dijkman, Nienke R. Biermasz, Camiel J. F. Boon, Alberto M. Pereira

**Affiliations:** ^1^Center for Endocrine Tumors, Division of Endocrinology, Department of Medicine, Leiden University Medical Center, Leiden, Netherlands; ^2^Department of Ophthalmology, Leiden University Medical Center, Leiden, Netherlands; ^3^Department of Ophthalmology, Academic Medical Center, University of Amsterdam, Amsterdam, Netherlands

**Keywords:** central serous chorioretinopathy, cortisol, cross-sectional study, Cushing’s syndrome, hypercortisolism, hypothalamic–pituitary–adrenal axis, psychosocial stress

## Abstract

**Objective:**

Central serous chorioretinopathy (CSC), a specific form of macular degeneration, has been reported as presenting manifestation of Cushing’s syndrome. Furthermore, CSC has been associated with both exogenous hypercortisolism and endogenous Cushing’s syndrome. It is important to know whether CSC patients should be screened for Cushing’s syndrome. Although hypothalamic-pituitary-adrenal (HPA) axis hyperactivity in CSC has been suggested, no detailed evaluation of the HPA axis has been performed in a large cohort of CSC patients. This study aimed to investigate whether Cushing’s syndrome prevalence is increased among chronic CSC (cCSC) patients and whether detailed endocrinological phenotyping indicates hyperactivity of the HPA axis.

**Design:**

Cross-sectional study.

**Patients:**

86 cCSC patients and 24 controls.

**Measurements:**

Prevalence of Cushing’s syndrome, HPA axis activity.

**Results:**

None of the cCSC patients met the clinical or biochemical criteria of Cushing’s syndrome. However, compared to controls, HPA axis activity was increased in cCSC patients, reflected by higher 24 h urinary free cortisol, and accompanying higher waist circumference and diastolic blood pressure, whereas circadian cortisol rhythm and feedback were not different. Chronic CSC patients did not report more stress or stress-related problems on questionnaires.

**Conclusion:**

No case of Cushing’s syndrome was revealed in a large cohort of cCSC patients. Therefore, we advise against screening for Cushing’s syndrome in CSC patients, unless additional clinical features are present. However, our results indicate that cCSC is associated with hyperactivity of the HPA axis, albeit not accompanied with perception of more psychosocial stress.

## Introduction

Cushing’s syndrome is a rare disease characterized by excessive exposure to cortisol and is associated with both metabolic and behavioral abnormalities. The clinical manifestation may vary, and in addition to well-known features like facial rounding, truncal obesity, and dorsal fat pad (1), ophthalmological abnormalities also occur. We recently reported patients who developed visual symptoms caused by chronic central serous chorioretinopathy (cCSC) as presenting manifestation of Cushing’s syndrome (2).

Central serous chorioretinopathy (CSC) is a relatively common eye disease often affecting the macula, in which choroidal congestion, thickening, and hyperpermeability lead to retinal pigment epithelial damage and cause serous subretinal fluid accumulation. Persistent neuroretinal detachments in untreated cCSC may result in irreversible photoreceptor damage, which may lead to permanent visual loss and decreased quality of life (3, 4).

The association of CSC with both exogenous steroids and endogenous hypercortisolism has been reported (2, 5, 6). Although no data are available on the prevalence of CSC in patients treated with corticosteroids, up to 52% of CSC patients in different cohorts reported to use steroids during the active phase of disease (6, 7). Higher endogenous cortisol levels were reported in 30 patients with acute CSC (8), and 24 h urinary free cortisol (UFC) was higher among 16 patients with chronic CSC compared to controls (9). However, clinical characteristics, circadian tests, and cortisol feedback were not included in these studies, making it impossible to conclude on the prevalence of Cushing’s syndrome.

In addition, psychosocial stress has been described in relation to CSC. Different studies reported associations between psychosocial stressful events and CSC, especially in patients with poor coping mechanisms (10). People with type A personality characteristics have been suggested to be at higher risk for the development of CSC (11).

In view of the suspected relationship between overactivity of the hypothalamic–pituitary–adrenal (HPA) axis and CSC, a relevant question is whether CSC patients should be screened for Cushing’s syndrome. Therefore, we conducted a systematic screening for the presence of Cushing’s syndrome in a large cohort of cCSC patients, using detailed clinical and biochemical evaluation of the HPA axis, and compared the latter to a control group. Furthermore, perceived stress was evaluated using validated questionnaires.

## Materials and Methods

### Study Design

Cross-sectional study with the following key objectives: to assess the prevalence of Cushing’s syndrome in cCSC patients and to assess whether cCSC is associated with hyperactivity of the HPA axis. If this second aim was confirmed, we aimed to explore the association between HPA axis hyperactivity and psychosocial stress in cCSC.

### Study Population

Eighty-six consecutive cCSC patients, who were followed at the Department of Ophthalmology at our tertiary referral center, were screened. The cCSC diagnosis had been confirmed by fundoscopy, digital color fundus photography (Topcon Corp., Tokyo, Japan), fundus autofluorescence (Spectralis HRA + OCT; Heidelberg Engineering, Heidelberg, Germany), spectral-domain optical coherence tomography (Spectralis HRA + OCT), fluorescein angiography (Spectralis HRA + OCT), and indocyanine green angiography (Spectralis HRA + OCT), according to current standard (5, 12–16). Patients diagnosed with acute CSC (focal leakage spot or a smokestack pattern on fluorescein angiography) were excluded (5, 12–16). No evidence of other retinal diagnoses had to be present.

Other exclusion criteria possibly affecting the evaluation of the HPA axis were use of corticosteroids/sleep medication prior to the development or during the time course of cCSC, excessive alcohol intake (>21 U/week), nightshift work, or traveling from another time zone in the 6 weeks before evaluation.

We also performed tests for hypercortisolism in a set of gender-matched controls. Thirty-eight healthy subjects responded to advertisements. Fourteen were excluded based on criteria described below. A total of 24 healthy gender-matched control subjects were eligible for inclusion (inclusion period: September 2015 to December 2016). Exclusion criteria were (familial) history of eye diseases/visual problems, psychiatric diseases, or chronic physical diseases possibly influencing endocrinological screening, corticosteroids/antidepressants/sleep medication use, excessive alcohol intake (>21 U/week), recent weight loss/gain of >10%, and working nightshifts or traveling from another time zone in the 6 weeks before evaluation.

Written informed consent was obtained from all participants and approval of the institutional review board and the ethics committee was obtained (NL50816.058.14).

### Endocrinological Evaluation

Screening was performed including a detailed medical history, complete physical examination, and biochemical analysis. The physical examination consisted among others of the evaluation of clinical Cushing stigmata and was performed by two physicians (FH/MB).

For evaluating HPA axis activity, all three commonly available screening tests were performed: UFC in two 24 h urine samples, midnight salivary cortisol (mSC), and 1 mg dexamethasone overnight suppression test. Healthy controls were subjected to one 24 h urine and one midnight saliva collection only. In case of deviant test results, participants were re-tested to exclude relevant pathology. The first test results were included in the analysis. UFC (82 patients and all controls) was analyzed using an in-house LC-tandem MS method, calibrated using Cerilliant certified reference material C-106, cortisol 1 mg/ml in methanol. The analytical variation was between 6.5 and 5% for urine cortisol levels between 50 and 900 nmol/L. Cortisol levels below 150 nmol/24 h were considered normal. Serum (81 patients) and salivary cortisol (82 patients and 23 controls) were analyzed using a Roche ECLIA Cortisol assay (second generation) on a Modular E170 immunoanalyser (Roche Holding AG, Basel, Switzerland). Analytical variation ranged between 10.1 and 1.9% for serum cortisol levels between 3.6 and 1,660 nmol/L and between 14.2 and 2.5% for saliva cortisol levels between 2.6 and 78 nmol/L. Cortisol levels below 1.5 nmol/L could not be determined. In midnight saliva, cortisol levels below 5.7 nmol/L were considered normal. The cutoff limit for the dexamethasone suppression test was 50 nmol/L (17).

### Questionnaires

#### Perceived Stress Scale (PSS)

The PSS developed by Cohen et al. was designed to measure the intensity of perceived stress and considers the degree to which individuals experience their lives as unpredictable, uncontrollable, and overloading (18). The original scale contained 14 items, but its creators refined it to 10 items, of which four are positively and six are negatively phrased (19). Items are coded from 0 to 4 and summed to compute a total score. Higher scores indicate greater perceived stress. Scores around 13 on the PSS are considered average, whereas high stress groups have reported scores of approximately 20 points (19).

#### Stress Thermometer

A visual analog scale was designed by the authors to measure the amount of stress experienced in the week before evaluation. Individuals rate their amount of stress on a scale from 0 to 10, with 0 indicating “no stress at all” and 10 indicating “the highest possible amount of stress.”

#### Insomnia Severity Index

This seven-item scale assesses self-reports of insomnia symptoms over the last 2 weeks. The items are scored on a scale from 0 to 4. Total scores of 0 to 7 are categorized as “no insomnia,” scores from 8 to 14 are considered to indicate “sub-threshold insomnia,” scores from 15 to 21 are indicative of “moderate insomnia,” and scores from 22 to 28 are considered “severe insomnia” (20).

#### Brugha Questionnaire on Life Events

This list to assess the occurrence of stressful events includes 12 life events that were found to have long-term negative effects on most people who experience them. Participants indicate whether certain events have occurred to them during the past year or earlier in their lives (21).

### Statistical Analysis

Based on data derived from a recent study by Aranda and colleagues (22), a power calculation was performed on the difference in 24 h UFC deemed relevant to detect (20 nmol/24 h). To detect such a difference (with power 80% and alpha 0.05), a sample size of 54 cCSC patients and 18 healthy controls would suffice.

Data were analyzed using SPSS Statistics (version 23; IBM Corp., Armonk, NY, USA). Data were presented as mean and SD, unless mentioned otherwise. The primary analyses comprised: (1) prevalence of Cushing’s syndrome in cCSC and (2) comparison of biochemical results between cCSC patients and healthy controls. Because the majority of cCSC patients were males (in line with other cohorts described in literature), a male-only sensitivity analysis was performed. Mean and SD scores for each questionnaire were calculated.

Normality of data was tested using the Shapiro–Wilk test. All normally distributed data were analyzed using independent sample *t*-tests. Data with a non-normal distribution were analyzed by means of nonparametric independent sample tests. The two groups were compared using a general linear model, correcting for potential confounders such as age, waist–hip ratio, and waist circumference. Associations were assessed using linear regression analyzes. The level of significance was set at *P* = 0.05. For the analysis of the questionnaires, the level of significance was set at *P* = 0.01 to correct for multiple testing.

After reassessment of the retinal imaging by two independent ophthalmologists, five patients considered to have less typical cCSC findings on imaging were excluded from analysis. Moreover, an analysis excluding outliers (*n* = 1) was performed. All results are described below.

## Results

### Baseline Characteristics

Eighty-six cCSC patients (77 males) and 24 healthy controls (19 males) were included (Table 1). The gender distribution was in line with available literature (5, 15). The mean duration of disease at the time of evaluation was 3.86 years (range 0.17–37.06). Fifty-eight patients had active CSC (presence of subretinal fluid) at the moment of screening.

**Table 1:** Clinical characteristics of participants.

	cCSC patients (*n* = 86)	Controls (*n* = 24)	*P* value
Age, years	48.74 (10.84)	41.08 (13.08)	0.004
Sex, male/female	77/9	19/5	0.182
Duration of cCSC disease, years (range)	3.86 (0.17–37.06)	–	–
History of hypertension, *n* (%)	23 (26.7%)	1 (4.2%)	0.023^a^
History of diabetes mellitus, *n* (%)	6 (7.0%)	0 (0.0%)	0.336
History of dyslipidemia, *n* (%)	18 (20.9%)	1 (4.2%)	0.068
History of psychiatric disorders,^b^*n* (%)	16 (18.6%)	1 (4.2%)	0.113
History of thromboembolic events, *n* (%)	0 (0%)	0 (0%)	–
History of cardiac events,^c^*n* (%)	5 (5.9%)	2 (8.3%)	0.648
History of sexual disorders,^d^*n* (%)	19 (22.1%)	1 (4.2%)	0.069
Systolic blood pressure, mmHg	135.41 (16.64)	129.75 (12.41)	0.143
Diastolic blood pressure, mmHg	82.94 (10.30)	77.29 (12.36)	0.006
Body mass index	26.15 (3.59)	24.92 (3.14)	0.096
Waist circumference, cm	92.74 (11.07)	86.42 (9.28)	0.011
Waist–hip ratio	0.95 (0.07)	0.90 (0.06)	0.003
Moon face, *n* (%)	1 (1.2%)	0 (0.0%)	1.000
Dorsal fat pad, *n* (%)	1 (1.2%)	0 (0.0%)	1.000
Purple striae, *n* (%)	0 (0.0%)	0 (0.0%)	–
Muscle weakness, *n* (%)	3 (3.5%)	0 (0.0%)	1.000
Active skin infections, *n* (%)	2 (2.3%)	0 (0.0%)	1.000
Hematomas, *n* (%)	3 (3.5%)	1 (4.2%)	1.000
Ankle edema, *n* (%)	2 (2.3%)	0 (0.0%)	1.000

*Data are presented as mean (SD) or as numbers, unless specified otherwise*.

*^a^Not statistically significant after correction for age*.

*^b^Consisting of depression, anxiety or panic disorder, posttraumatic stress disorder, burn-out, alcohol abuse, and schizophrenia*.

*^c^Consisting of myocardial infarction, endocarditis, and atrial fibrillation*.

*^d^Consisting of impotence, hirsutism, menstrual cycle disorders, and loss of libido*.

*cCSC, chronic central serous chorioretinopathy*.

There was no difference in gender distribution or body mass index between the two groups. Patients were 7.5 years older than controls.

### Clinical Evaluation

None of the cCSC patients presented with a combination of clinical signs and symptoms typical for Cushing’s syndrome. Hypertension was reported by 27% of patients and one control (4%, *P* = 0.023). In addition, cCSC patients had a higher prevalence of other comorbidities, e.g., dyslipidemia and psychiatric disorders (see Table 1). Waist circumference, waist–hip ratio, and diastolic blood pressure were higher in patients compared to controls, despite a higher prevalence of ongoing antihypertensive medication use in the patient group. These differences remained significant after adjustment for age. Characteristic Cushing features were rare among cCSC patients.

### Hormonal Evaluation

#### Clinical Evaluation of Patients

None of the cCSC patients had Cushing’s syndrome, but several patients demonstrated an abnormally high cortisol value in one or more of the screening tests (Table 2). Increased UFC (>150 nmol/24 h, average of two portions) was present in seven patients, in whom repeated testing revealed normal values. Increased mSC levels (>5.7 nmol/L, average of two portions) was observed in three patients, which normalized upon retesting in two and persisted to be slightly elevated in one patient. Insufficient suppression after dexamethasone was observed in four patients. Retesting revealed normal test results in one patient. In the absence of other biochemical and clinical features of Cushing’s syndrome, we concluded that the abnormal test results in the other patients were likely due to intervening medication (antidepressants, gonadotropin-releasing hormone analogs, covert stimulant use). Furthermore, normal lipid profiles, no elevated inflammation parameters, and no hypokalemia were detected (data not shown).

**Table 2:** Biochemical characteristics of participants.

	cCSC patients	Controls	*P* value
Urinary free cortisol, nmol/24 h^a^	83.99 (49.04)	51.55 (28.49)	0.000
Detectable midnight salivary cortisol,^a^^,^^b^ %	24.4	60.9	0.002
Serum cortisol after 1 mg Dexa, μmol/L^a^	0.032 (0.047)	–	–

	**cCSC patients with active disease**	**cCSC patients with inactive disease**	

Urinary free cortisol, nmol/24 h^a^	78.44 (38.63)	95.30 (64.76)	0.524
Detectable midnight salivary cortisol,^a^ %	27.3	18.5	0.428
Serum cortisol after 1 mg Dexa, μmol/L^a^	0.028 (0.045)	0.031 (0.041)	0.855

*Data are presented as mean (SD) or as numbers, unless specified otherwise*.

*^a^Number of participants*.

*Urinary free cortisol: 82 cCSC patients (55 active cCSC patients, 27 inactive CSC patients) and 24 controls*.

*Midnight salivary cortisol: 82 cCSC patients (55 active cCSC patients, 27 inactive CSC patients) and 23 controls*.

*Serum cortisol after 1 mg dexamethasone: 55 active cCSC patients, 26 inactive CSC patients*.

*^b^>1.5 nmol/L*.

*cCSC, chronic central serous chorioretinopathy; Dexa, dexamethasone*.

#### Comparison with Healthy Controls

**Figure 1:**
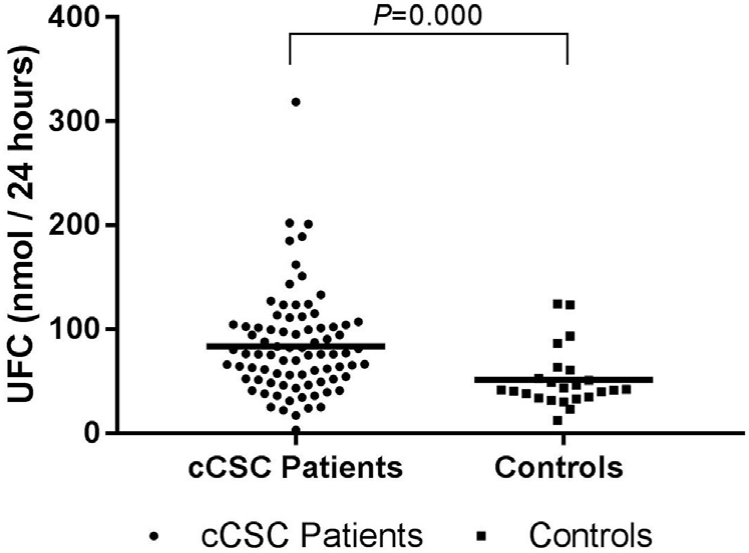
UFC levels in cCSC patients and healthy controls. Notes: Data presented as individual values and mean. Patients *n* = 82; controls *n* = 24. Abbreviations: UFC, urinary free cortisol; cCSC, chronic central serous chorioretinopathy.

Mean UFC levels were higher in cCSC patients, compared to controls (Figure 1). This difference remained after correction for age (*P* = 0.001), age, and waist–hip ratio (*P* = 0.002), age and comorbidities (e.g., psychiatric disorders, diabetes mellitus, hypertension, obesity; *P* = 0.011), and when males were evaluated solely (*P* = 0.001).

Nonparametric analysis revealed that non-detectable mSC was present in 76% of cCSC patients compared to 39% of healthy controls (*P* = 0.002), with a similar difference in a male-only analysis (72 versus 33%, *P* = 0.001). Three patients (4%) showed abnormally elevated mSC, compared to two controls (9%). The other participants’ mSC levels were between 1.5 and 5.7 nmol/L.

#### HPA Axis at Different cCSC Disease Stages

**Figure 2:**
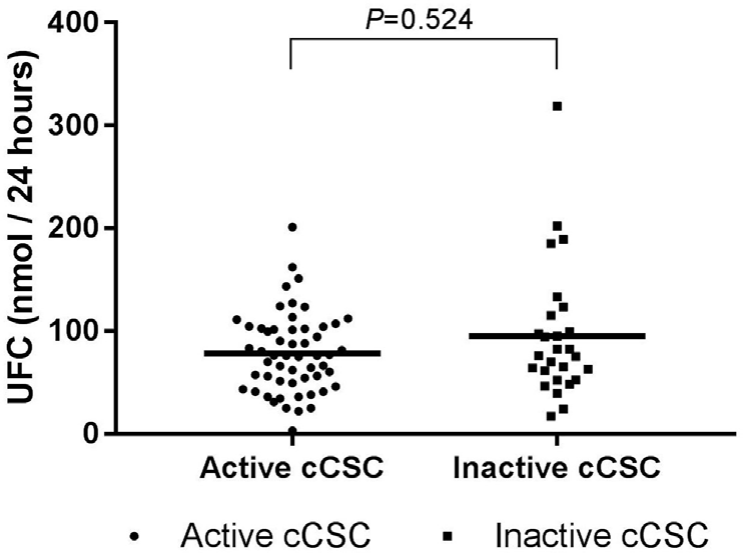
UFC levels in active cCSC patients and inactive cCSC patients. Data presented as individual values and mean. Active cCSC patients *n* = 55; inactive cCSC patients *n* = 27. Abbreviations: UFC, urinary free cortisol; cCSC, chronic central serous chorioretinopathy.

Hypothalamic–pituitary–adrenal axis activity was not different between patients with active and inactive cCSC. Mean UFC levels were 78.44 (SD 38.63) versus 95.30 (SD 64.76), respectively (*P* = 0.524, Figure 2), and mSC was detectable in 27% of patients with active disease versus 19% of patients with inactive disease (*P* = 0.386).

The exclusion of five atypical cCSC patients or the exclusion of one outlier in UFC did not affect any of the described results. Clustered analysis did also not significantly change the aforementioned results (data not shown).

### Questionnaire Analysis

#### Perceived Stress Scale

Chronic CSC patients (*n* = 81, 94%) reported a mean total score of 12.95 (SD 5.82, range 0–30). After correction for multiple testing, no significant difference in PSS score was found between cCSC patients with active disease compared to inactive patients (mean 11.89 versus 15.07, *P* = 0.019). Furthermore, no association was found between UFC level and the total score in patients (β = 2.04, *P* = 0.032, *R*^2^ = 0.06).

#### Stress Thermometer

Eighty-three cCSC patients (96%) scored their amount of experienced stress in the week prior to evaluation, reporting a mean score of 4.4 (range 0–10). In addition, no differences were found when active patients were compared to patients with inactive disease, and no association between UFC level and score on this scale was found (β = 0.71, *P* = 0.742, *R*^2^ = 0.001).

#### Insomnia Severity Index

Total scores were calculated for 83 cCSC patients (mean 6.54, range 0–24). The mean score was categorized as “no clinical significant insomnia.” When insomnia was scored as a “yes or no” variable, 11% of patients scored either moderate or severe insomnia. There was no difference in presumed insomnia between active patients and inactive patients, and no association between UFC levels and Insomnia Severity Index scores was found (β = −0.97, *P* = 0.378, *R*^2^ = 0.010).

#### Brugha Questionnaire on Life Events

Thirty out of 83 patients (36%) reported serious life events in the past year. Twenty-four hour UFCs were not higher in these patients compared to the patients with no serious life event in the preceding year. Disease activity did not affect the report of serious life events in the past year (*P* = 1.000). The type of life events experienced, however, was different, with active patients reporting more experiences with serious illness or violence of a near relative, whereas inactive patients more often reported the same experiences earlier in live.

#### Associations of Questionnaire Outcomes and Cortisol

Analyses performed with exclusion of the five atypical cCSC patients and analyses with exclusion of the UFC outlier did not significantly change the aforementioned results.

## Discussion

This is the first study that systematically evaluated various aspects of the activity of the HPA axis in a large cohort of cCSC patients. Whereas we did not find any case of Cushing’s syndrome, the activity of the HPA axis appeared to be increased in cCSC patients, without disruption of circadian rhythm. This was reflected by significantly higher 24 h UFCs, increased waist circumference, and diastolic blood pressure, but normal mSC levels. Our study demonstrates that systematic screening of all cCSC patients for the presence of Cushing’s syndrome is not indicated.

In the present study, we have screened a large patient cohort in detail, combining all currently available biochemical screening tests with a detailed clinical phenotyping. We found significantly higher 24 h UFC levels in cCSC patients, albeit within the normal reference range, with preservation of normal diurnal rhythmicity. Elevated UFC levels have been reported previously in a small cohort of acute CSC patients during hospital admission, when compared to patients with acute retinal detachment (8), and in a small cohort of cCSC patients that were compared to age- and sex-matched controls (9), though data on demographics of these participants were lacking.

In contrast to earlier studies suggesting an association between cCSC and psychosocial stress, we did not find a clear relationship between cCSC activity and stress, based on the results of four stress questionnaires. In addition, no association was found between HPA axis activity and psychosocial stress. A critical evaluation of the available literature does not support a clear association between cCSC and stress: Conrad et al. demonstrated no increased exposure to critical life events in 30 CSC patients and reported other findings suggestive of difficulties in emotional regulation (23). Other studies reported an association between stress, severe stressful events, and CSC, especially in patients with poor coping mechanisms (10, 24, 25), but the provided information on how stress was measured was very limited, circumstantial, or even absent. Our patients scores (on the PSS) did not differ from reported average scores and were not comparable with scores reported by high stress groups (19). In addition, our patients with active cCSC reported no difference in experienced life events, insomnia (as an expression of stress) or perceived stress on two different scales, indicating that cCSC activity is not associated with psychosocial stress.

Both endogenous hypercortisolism and exogenous administration of corticosteroids are related to CSC (2, 5, 6, 9). Occurrence of one or more episodes of CSC has previously been described in 5% of 60 patients with active endogenous hypercortisolism. All these CSC patients had been diagnosed with pituitary adenoma (26). Fundus characteristics resembling CSC have also been reported in patients with Cushing’s disease (27). Moreover, in a patient with hypercortisolism due to adrenocortical carcinoma, bilateral CSC has been found (28). Several underlying mechanisms have been hypothesized. Endogenous hypercortisolism increases platelet aggregation leading to microthrombi and increased blood viscosity, which could be of importance in the pathogenesis of CSC (29). Hypercortisolism has also been associated with choroidal fragility and hyperpermeability (30). Moreover, increased transcription of adrenergic receptors has been correlated with CSC (31). In addition, a role for the mineralocorticoid pathway has been suggested by recent studies in rats and by findings in CSC patients treated with mineralocorticoid receptor antagonists (eplerenone or spironolactone) (15, 32). Both glucocorticoids and mineralocorticoids activate the mineralocorticoid receptor expressed on choroidal endothelial cells. Activation of the mineralocorticoid receptor, *via* upregulation of the endothelial vasodilatory calcium-dependent potassium channel KCa2.3 by hyperpolarization of these endothelial cells and of smooth muscle cells, has been suggested to lead to vasodilation (15).

Our study also has limitations. The cross-sectional character does not allow drawing conclusions on any causal relationship. Furthermore, a reversed causation (cCSC as a trigger for activation of the HPA axis) seems to be less likely in light of the currently available literature, yet is not ruled out. Also, the number of healthy control subjects recruited *via* advertisements was limited, and because our study was not powered for the questionnaire outcomes, we did not compare patient and control data. Only one 24 h urine sample was collected by healthy controls. Volume and creatinine level analyses confirmed adequate collection of these single samples. The fact that 24 h UFC is higher in the presence of equal results of mSC can very well be explained by an increased activity of the HPA axis with preservation of normal diurnal rhythmicity (in contrast to the “autonomous” cortisol secretion that is characterized by loss of diurnal rhythmicity). The absence of associations between UFC level and either cCSC activity or outcomes of stress questionnaires in our study may appear to be contradictory to the conclusion that the HPA axis is more activated in cCSC patients. Nonetheless, one should keep in mind that there is a wide individual variation in normal cortisol levels and in cortisol receptor activation thresholds, leading to different thresholds for the development of cortisol-related symptoms and pathology. Together, this may explain why the HPA axis could still be activated in cCSC patients despite the absence of an association between UFC and cCSC activity or questionnaire outcomes in our patient population.

Although CSC has been described to be a presenting symptom of Cushing’s syndrome and these diseases are known to sporadically co-exist (2), our results argue against screening for endogenous hypercortisolism in all cCSC patients. Since the interpretation of the available biochemical screening tests in light of the clinical features is challenging and in order to minimize the risk of false positive test results, screening should be reserved for those cCSC patients in whom clinical signs or symptoms raise suspicion of Cushing’s syndrome. Only then patients should be referred to an endocrinologist for evaluation of the HPA axis. In dealing with Cushing’s syndrome patients, endocrinologists also need to be aware of the potential coexistence of CSC.

In conclusion, systematic screening of all patients with cCSC for Cushing’s syndrome is not indicated. However, the activity of the HPA axis appears to be increased, with preservation of circadian rhythm. Finally, in contrast to earlier ideas, we did not find obvious associations between cCSC, cCSC activity, and psychosocial stress. The observed hyperactivity of the HPA axis confirms the previously reported association between cortisol and CSC and merits further studies to unravel the underlying pathophysiological mechanisms.

## Ethics Statement

All subjects gave written informed consent in accordance with the Declaration of Helsinki. The protocol was approved by the institutional review board and the ethics committee (NL50816.058.14).

## Author Contributions

All authors listed have made a substantial, direct, and intellectual contribution to the work and approved it for publication. FH and MB collected the data. FH and ED wrote the paper and designed the figures. AP, CB, NB, OD, and GD evaluated the paper.

## Conflict of Interest Statement

The authors declare that the research was conducted in the absence of any commercial or financial relationships that could be construed as a potential conflict of interest.
